# Protective effect of nebivolol on rat ovary exposed to deltamethrin toxicity

**DOI:** 10.1590/acb385423

**Published:** 2023-10-23

**Authors:** Serap Otçu, Engin Deveci, Çağdaş Özgökçe, Görkem Tutal Gürsoy, Mehmet Cudi Tuncer

**Affiliations:** 1 Health Sciences University – Diyarbakır Gazi Yaşargil, Training and Research Hospital – Department of Obstetrics and Gynecology – Diyarbakır – Turkey.; 2 Dicle University – Medical School – Department of Histology and Embryology – Diyarbakır – Turkey.; 3 Zeynep Kamil Hospital – Department of Obstetrics and Gynecology – Perinatology Department – Istanbul – Turkey.; 4 Healt Ministry of Türkiye Republic – Ankara Bilkent City Hospital – Department of Neurology – Ankara –Turkey.; 5 Dicle University – Medical School – Department of Anatomy – Diyarbakır – Turkey .

**Keywords:** Ovary, Nebivolol, Proliferating Cell Nuclear Antigen, Tumor Necrosis Factor-alpha

## Abstract

**Purpose:**

We aimed to investigate the antioxidant activity of nebivolol against possible damage to the ovarian tissue due to the application of deltamethrin as a toxic agent, by evaluating histopathological proliferating cell nuclear antigen (PCNA) and tumor necrosis factor-alpha (TNF-α) signal molecules immunohistochemically.

**Methods:**

The animals were divided into three groups as control, deltamethrin and deltamethrin + nebivolol groups. Vaginal smears were taken after the animals were mated and detected on the first day of pregnancy. After the sixth day, deltamethrin (0.5 mL of 30 mg/kg BW undiluted ULV), and 2 mL of sterile nebivolol solution were administered intraperitoneally every day for 6-21 periods. After routine histopathological follow-up, the ovarian tissue was stained with hematoxylin and eosin stain.

**Results:**

Control group showed normal histology of ovarium. In deltamethrin group, hyperplasic cells, degenerative follicles, pyknotic nuclei, inflammation and hemorrhagic areas were observed. Nebivolol treatment restored these pathologies. Deltamethrin treatment increased TNF-α and PCNA reaction. However, nebivolol decreased the expression.

**Conclusions:**

It was thought that deltamethrin toxicity adversely affected follicle development by inducing degeneration and apoptotic process in preantral and antra follicle cells, and nebivolol administration might reduce inflammation and slow down the apoptotic signal in the nuclear phase and regulate reorganization.

## Introduction

Deltamethrin is a type II synthetic pyrethroid effective on insects and parasites^1^^.^ 2. Due to the widespread use of pyrethroids in agricultural countries^3^, its contamination is a worldwide problem. The presence of deltamethrin, especially in unhygienic drinking water, still threatens human life^4^. Deltamethrin’s relationship with toxicity and oxidative stress is important. Oxidative damage caused by deltamethrin toxicity occurs with the body’s antioxidant state and differences in oxidative signaling pathways^5^. Oxidative stress injury occurs through the generation of reactive oxygen species (ROS) by deltamethrin^6^.

Proliferative cell nuclear antigen is a nuclear protein that regulates cell morphology and is involved in DNA synthesis^7^,^8^. The proliferating cell nuclear antigen (PCNA) level increases as the cell turns from the G1 phase to the S phase and then to the M phase, respectively, before DNA synthesis takes place^9^. Relationship of PCNA to cell proliferation activity is important, because it has been reported to be used as a marker of cell proliferation for the differentiation of healthy and tumor tissues.

**Figure 1:**
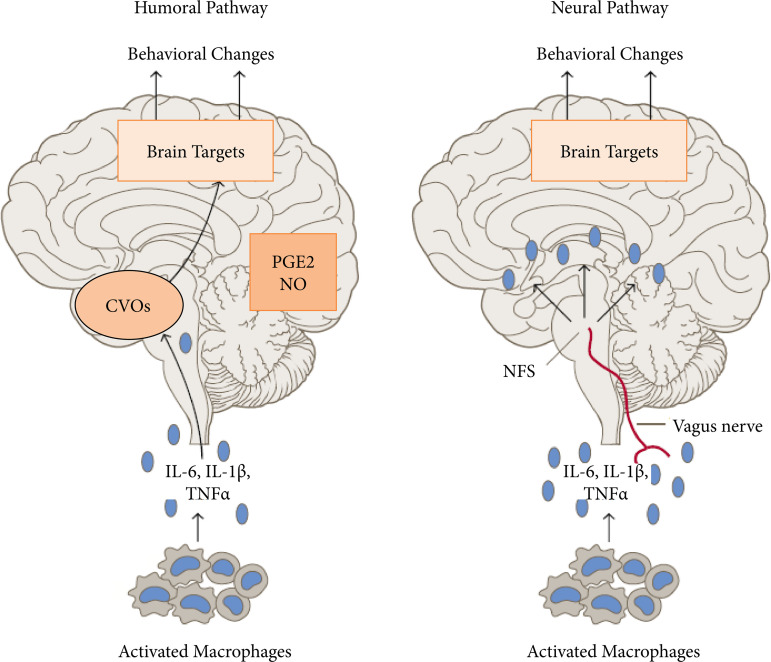
Appearance of humoral and neural pathways of cytokines including TNF-α from macrophages.

CVO: circumventricular organ; PGE_2_: prostaglandin E2; NO: nitric oxide; NTS: the nucleus of *tractus solitarii*; IL: interleukin; TNF-α: tumor necrosis factor-alpha. Source: https://mavink.com/explore/Macrophage-Release-Cytokines

During the inflammatory reaction processes, tumor necrosis factor-alpha (TNF-α) becomes active as a result of the migration of leukocytes, inflammatory chemokines and cytokines to the tissue damage area and shows its effect in the damaged area. Oxidative stress is known to increase inflammation in ischemia and reperfusion (I/R) injury. Increases in the level of TNF-α, an important proinflammatory cytokine, occur at the injury site due to migration of reactive cells to the injury site, tissue disruption^10^,^11^. It is known that cytokines from macrophages reach the brain via the vascular and vagus nerves. In summary, it is known that access to the brain is provided in two different ways. It has been reported that it allows this transition in areas that do not have a blood-brain barrier. Secondary messenger structures such as prostaglandin and nitric oxide transmit the impulse to the nucleus of the tractus solitarius in the brainstem via the vagus nerve. (Fig. 1).

**Figure 2:**
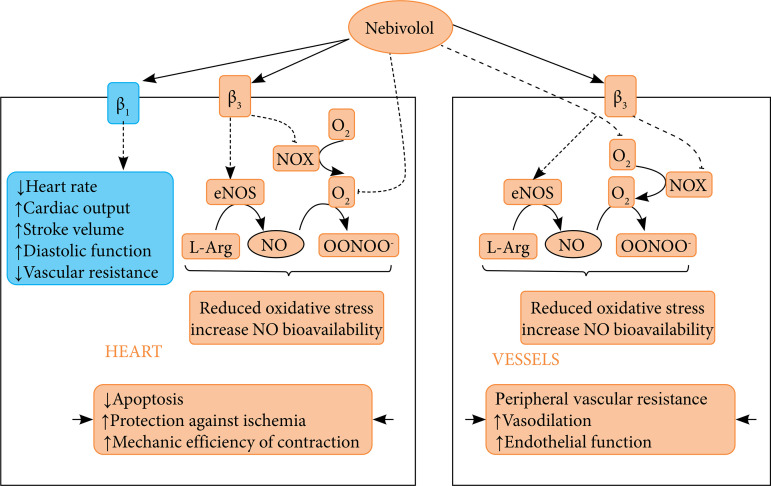
View of the mechanism of action of nebivolol on the heart and blood vessels.

NADPH: reduced nicotinamide adenine dinucleotide phosphate; NOX: NADPH oxidase; eNOS: endothelial nitric oxide synthase; NO: nitric oxide; ( stimulation;  inhibition). Source: Elaborated by the authors.

Nebivolol, a vasodilating beta-blocker, has been reported to have beta-adrenergic blocking activity. It has been stated that it protects left ventricular functions by decreasing heart rate, increasing cardiac output and stroke volume in patients with hypertension. However, nebivolol maintains health by reducing oxidative stress and increasing nitric oxide bioavailability through receptor-dependent inhibition of O_2_- and β3-adrenergic receptor-dependent reduced nicotinamide adenine dinucleotide phosphate (NADPH) oxidase and endothelial nitric oxide synthase degradation. In addition, they observed that nebivolol provides improvement at the vascular level by utilizing nitric oxide through β3-adrenergic receptors in vascular system disorders. It was observed that nebivolol inhibited NADPH oxidase activity in hypertension models created in different experimental animals^12^-^14^. For this reason, it has been reported that nebivolol’s endothelial nitric oxide synthase stimulating effect and ROS abolition effects have cardiac positive effects apart from its β1-antagonistic effect. (Fig. 2).

It has been suggested that nebivolol has an antioxidant structure, as well as protective effects on cardiac output, and has positive effects on lipid and carbohydrate mechanisms. It has been also observed that the incidence of neuronopathic, cardiac, erectile dysfunction and respiratory problems associated with the use of nebivolol is low^15^,^16^.

We aimed to investigate the antioxidant activity of nebivolol against possible damage to the ovarian tissue due to the application of deltamethrin as a toxic agent, by evaluating biochemical, histopathological PCNA and TNF-α signal molecules immunohistochemically.

## Methods

### Animals and experimental design

In this experimental study, female Wistar rats weighing 200–250 g were used for examination. Animals were obtained from Dicle University Faculty of Medicine Animal Care Unit, Diyarbakır, Turkey. They were housed at 25°C and 45–55% humidity with a 12/12-hour dark and light cycle. Animals were fed a pellet diet and water *ad libitum*, following the Laboratory Animal Care Principles (NH publication no. 85-23, revised 1985) for animal care during experiments.

Deltamethrin (C22H19Br2NO3) (> 99% pure) was dissolved in corn oil. CAS, chemical name a-cyano-3-phenoxybenzyl (1R, 3R)-3-(2,2-dibromovinyl)-2,2 dimethyl cyclopropane-carboxylate, is manufactured by El Afrane-1009, Elouardia, a Tunisian fertilizer company.

After the mating of the female animals, vaginal smear was taken, and the first day of pregnancy was detected. Deltamethrin and nebivolol were made between 6–21 days after the sixth day. Immediately after birth, pup rats were taken and divided into groups. Wistar rats (n = 30) were randomly divided into three groups:

- Control group (n = 10): treated with 0.5 mL corn oil by oral gavage;
- Deltamethrin group (n = 10): deltamethrin was given (0.5 mL of 30 mg/kg BW undiluted ULV) (a fifth of LD_50_)^17^.
- Deltamethrin + nebivolol group (n = 10): after administration of deltamethrin, nebivolol was given. It was prepared from 5 mL of nebivolol tablets (Vasoxen, Menarini Group, I. E. Ulagay, Germany) by pulverizing and dissolving in distilled water to obtain 0.017 mg / mL solution (X). Sterile nebivolol solution was administered intraperitoneally with 2 mL every day for two weeks.

### Histopathologic analysis

Ovarian tissues were fixed with zinc-Formalin solution (catalog no. Z2902, Sigma-Aldrich, St. Louis, MO, United States of America) and washed under tap water by 10 minutes. Tissues were passed through ascending alcohol series for about 24 hours. Tissues were washed with xylene 2 × 15 minutes and incubated within paraffin wax. Five-μm sections were cut with microtome (catalog no. Leica RM2265, Wetzlar, Germany). Deparaffinized within xylene for 2 × 15 minutes, sections were brought to distilled water. Some of the sections were stained with routine hematoxylin and eosin (H&E). The rest were kept for immunohistochemical staining^18^.

### Immunohistochemical analysis

Ovarian tissues were brought to distilled water. Hydrogen peroxide solution (catalog no. TA-015-HP, Thermo Fischer, Fremont, CA, United States of America) were dropped on sections for 15 minutes. After washing in phosphate buffer solution (PBS) for 3 × 5 minutes, ultra V Block (catalog no. TA-015-UB, Thermo Fischer, Fremont, CA, United States of America) was applied to sections for 6 minutes. Sections were incubated with primary antibody TNF-α and PCNA (AFG Bioscientific, United States of America) at +4°C overnight. Sections were allowed to warm at room temperature for 30–60 minutes. Sections were washed with biotinylated secondary antibody (catalog no. TP-015-BN, Thermo Fischer, Fremont, CA, United States of America) for 10 minutes. Streptavidin-peroxidase (catalog no. TS-015-HR, Thermo Fischer, Fremont, CA, United States of America) was dropped onto sections for 10 minutes. Clearing with PBS, 3,3’-diaminobenzidine (DAB) (catalog no. TA-001-HCX, Thermo Fischer, Fremont, CA, United States of America) was used as chromogen. Sections were counter stained with Gill hematoxylin (catalog no. 105174, Sigma-Aldrich, St. Louis, MO, United States of America) and mounted with entellan (catalog no. 107961, Sigma-Aldrich, St. Louis, MO, United States of America). Slides were analyzed with Zeiss Imager A2 Zen 3.0 software (Germany, Carl-Zeiss-Straße, Oberkochen, Germany) and photomicrographed^19^.

### Statistical analysis

The data were recorded as arithmetic mean ± standard deviation with mean rank value. Statistical analysis was done using the IBM Statistical Package for the Social Science 25.0 software (IBM, Armonk, New York, United States of America). Kruskal–Wallis’ test was used for multiple comparisons with post hoc Bonferroni’s test. Statistically significant level was accepted as p < 0.05.

## Results

Statistical analysis of biochemical was shown in Table 1. Granular cell degeneration, vascular dilatation and congestion, inflammation, PCNA and TNF-α expression were increased in del group compared to control and deltamethrin + nebivolol groups, and the increase was statistically significant. Granular cell degeneration, vascular dilatation and congestion, inflammation, PCNA and TNF-α expression were decreased compared to del group, and the values were close to control group (Table 1).

**Table 1:** Histopathological (granular cell degeneration, vascular dilatation and congestion, inflammation) and immunohistochemical scores (PCNA and TNF-α expression) of control, deltamethrin and deltametrin + nebivolol groups.

Parameters	Groups	n	Mean + SD	Mean rank	p-value
Granular cell degeneration	Control	10	0.70 ± 0.67	6.40	*p < 0.001 **p < 0.032
	Deltamethrin	10	3.80 ± 0.42	24.10	
	Deltametrin + nebivolol	10	2.40 ± 1.07	16.00	
Vascular dilatation and congestion	Control	10	0.50 ± 0.53	9.50	*p = 0.002 **p = 0.001
	Deltamethrin	10	3.60 ± 0.52	25.50	
	Deltametrin + nebivolol	10	0.80 ± 0.79	11.50	
Inflammation	Control	10	0.50 ± 0.53	9.25	*p < 0.001 **p < 0.001
	Deltamethrin	10	3.70 ± 0.48	25.50	
	Deltametrin + nebivolol	10	0.80 ± 0.63	11.75	
PCNA expression	Control	10	0.60 ± 0.52	8.60	*p = 0.021 **p = 0.001
	Deltamethrin	10	3.40 ± 0.84	25.20	
	Deltametrin + nebivolol	10	1.10 ± 0.74	12.70	
TNF-α expression	Control	10	2.20 ± 0.79	11.30	*p = 0.012 **p = 0.008
	Deltamethrin	10	3.40 ± 0.52	22.80	
	Deltametrin + nebivolol	10	2.30 ± 0.82	12.40	

* Control *vs*. deltamethrin;

** deltamethrin *vs*. deltamethrin + nebivolol;

SD: standard deviation; TNF-α: tumor necrosis factor-alpha; PCNA: proliferating cell nuclear antigen. Source: Elaborated by the authors.

## Histopatologic findings

**Figure 3:**
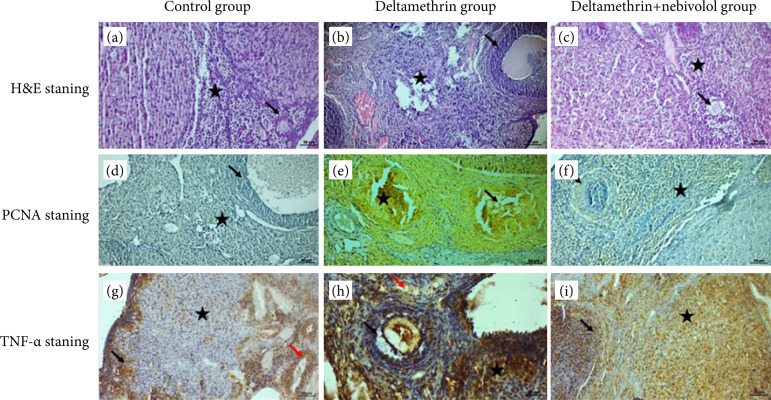
Cross-sections of all groups of H&E, PCNA and TNF-α staining. **(a)** In the control group sections, cuboidal cells in the ovarian germinal epithelium, oval and nuclei of granular cells around the preantral and antral follicles were observed to be rich in chromatin. It was observed that the connective tissue fibers, in which the connective tissue cells were solitary distributed in the stromal area outside the follicular area, were arranged parallel to each other, and the lumens of the blood vessels were regular and normal. Preantral and antral follicles (arrow), connective tissue (star); **(b)** In the histopathological section of the deltamethrin applied group, hyperplasic cells in the ovarian germinal epithelium, degenerative changes in most of the preantral and antral follicle cells, pyknosis in some nuclei, and apoptotic-looking nuclei in the oocyte cells in the advanced follicular structures were observed. An increase in inflammatory cells was observed within the interfollicular area, while dilatation and hemorrhagic areas were observed in the blood vessels. Degenerative changes in the preantral and antral follicle cells (arrow), inflammatory cells (star), the blood vessels (red arrow); **(c)** In the deltamethrin + nebivolol group, degenerative changes in some of the antral follicle cells and a small number of inflammatory cell infiltration were observed in their peripheral parts. It was observed that the structure of the zona pellucida around the oocyte cell in the antral follicle was regular, and the granulosa cells were rich in chromatin. It was observed that dilatation and congestion in the blood vessels in the stromal region decreased. Degenerative changes in antral follicle cells (arrow), inflammatory cell infiltration (star), dilatation and congestion in the blood vessels (red arrow). **(d)** PCNA reaction was negative in the blood vessel endothelial cells in the connective tissue cells in the stromal area outside the follicle in the pre-antral and antral follicle cells in the control group sections. Negative PCNA reaction in the stromal area (star), pre-antral and antral follicle cells (arrow). **(e)** In the deltamethrin group, PCNA expression was found to be positive with an increase in follicular cells and cumulus cells due to degeneration in preantral and antral follicle cells. PCNA positive cells were detected in most of the stromal cells outside the follicular area. Positive PCNA expression in follicular cells (arrow) and in stromal cells (star). **(f)** In the deltamethrin + nebivolol applied group, it was observed that some of the granulosa cells in the preantral and antral follicles towards the outer part showed negative PCNA expression in the stromal area, where the PCNA reaction was positive. Negative PCNA expression in the preantral and antral follicles (arrow), positive PCNA reaction in stroma (star). **(g)** In the examination of the sections of the control group, TNF-α reaction was observed at a mild level in some of the preantral and antral follicle cells, while TNF-α reaction was observed in the macrophage cells around the blood vessels. TNF-α reaction in preantral and antral follicle cells (arrow), macrophage cells (star) and the blood vessels (red arrow). **(h)** In the deltamethrin applied group, a significant increase in TNF-α expression in follicular cells around the *corpus luteum*, and a degenerative change in the zono pellucida around the oocyte cells in the antral follicle cells, together with a positive TNF-α expression in the area where it clustered in cells, was observed. TNF-α expression in inflammatory cells around the blood vessels in the stromal area observed as positive. TNF-α expression in follicular cells (arrow), inflammatory cells (star), blood vessels (red arrow). **(i)** In the deltamethrin + nebivolol group, moderate TNF-α expression was positive in the follicle cells in the advanced stage of the corpus luteum, while TNF-α expression was mild due to decreased inflammation in the cells in the stromal region.

Figure 3 shows H&E (Figs. 3a–3c) and immunohistochemical (Figs. 3d–3i) staining.

H&E: hematoxylin and eosin; PCNA: proliferating cell nuclear antigen; TNF-α: tumor necrosis factor-alpha. Source: Elaborated by the authors. TNF-α expression in the follicles (arrow) and stromal region (star). Scale bar: 50 μm, magnification: 20x for all sections.

## Discussion

Deltamethrin metabolite is known to cause toxicity due to oxidative stress. Deltamethrin (10 μM) significantly increased ROS production in PC12 cells^20^. According to Kumar et al.^21^, 25 and 50 μM deltamethrin for 1 hour increased levels of ROS, which is a marker of apoptosis in thymocytes. In another study, deltamethrin (1 and 5 μM) induced cell death and ROS production in rat primary hepatocytes^22^. These studies demonstrate that deltamethrin could directly cause oxidative stress damage via ROS production. In a study of Issam et al.^23^, on male Wistar rats in order to investigate the toxic effect of deltamethrin given for 30, 45 and 60 days (2, 20 and 200 mg/kg BW, subcutaneous injection) has been shown to increase malondialdehyde (MDA) levels. Xu et al.^24^ found that deltamethrin (90 days, 1.02, 2.56 and 6.40 mg/kg BW, oral gavage) inhibited superoxide dismutase and catalase activities and induced MDA and protein carbonyl levels in rat liver. In female rats, 30, 45 and 60 days administered deltamethrin (0.003, 0.03 and 0.3 mg/kg BW, subcutaneous injection) lead to significant DNA damage^25^.

In primary hepatocytes isolated from male Wistar rats, deltamethrin induced necrotic damage and inflammatory response. Deltamethrin gives damage to several organs; for instance, a large number of degenerative cells, pyknotic nuclei, and apoptosis were observed in the hippocampus and cortex 24 and 48 hours after deltamethrin exposure in Sprague–Dawley rats with a single dose exposure (12.5 mg/kg BW, intraperitoneal injection)^26^. Deltamethrin induced nephrotoxicity in rats for four weeks (2 mg/kg BW, orally)^27^.

Exposure to deltamethrin (0, 2, 5, 10, 20 or 40 mg/kg BW) for seven days caused neurotoxicity and liver dysfunction with decreased ROS levels^28^. In rat tests, deltamethrin exposure caused sperm abnormalities and lipid peroxidation^29^. Apoptosis and oxidative stress signaling were observed in deltamethrin-induced nephrotoxicity^30^. Moreover, immunotoxicity induced by deltamethrin caused changes in thymocyte and splenocyte apoptosis and immune function^3^,^31^. In our study, the increase in inflammation and the increase in degeneration together with the changes in the vascular structure with the application of deltamethrin caused cell damage by significantly affecting the apoptotic signaling.

It has been stated that PCNA is a key factor in many basic cellular processes such as DNA replication, DNA repair, sister-chromatid cohesion, avoidance of DNA damage, and cell^32^. They showed that PCNA expression was not detected in rat ovaries, granulosa cells or oocytes in primordial follicles, but increased with the onset of follicle growth. Increased expression of PCNA in oocytes around the initiation of primordial follicle formation and two-thirds of oocytes die during primordial follicle formation^33^,^34^. It has been thought that PCNA has a role in the regulation of oocyte fate, which can be explained by death or survival to form primordial follicles. Deltamethrin applied ovarian section showed significant PCNA increase in follicular cells, cell apoptosis became evident in the nuclear phase. There are no studies on the adverse effects of deltamethrin on ovarian tissue. There are no studies evaluating both PCNA and TNF-α signaling molecules.

In this study, we think that nebivolol reduces deltamethrin toxicity, which increases the NO release mechanism from endothelial cells. It is known that nebivolol is a β1-adrenergic receptor antagonist with vasodilation effect on the cardiac system and antioxidant structure^35^. Nebivolol has resulted in a significant reduction of the atherosclerotic lesions by the elevated dietary cholesterol and an improvement of the endothelial function and reduced the enhanced expression of inflammatory and oxidative damage markers (macrophages, adhesion molecules, and oxidative epitopes)^36^,^37^. After the application of nebivolol, the degenerative signal due to the reduction of inflammation made a significant contribution to the arrest of the nuclear stage, and it also showed a significant effect in terms of the reorganization of stromal activity.

NO is a marker that determines the extent of vascular damage. It has been reported that NO causes intracellular morphological changes, and these effects are especially related to vascular, endothelial cell regeneration, and leukocyte and thrombocyte values. Many drugs show their therapeutic actions through the production of NO bioactivity^37^. Nebivolol exerts strong antioxidant effects by stimulating NO synthesis. A four-week treatment model with nebivolol has been reported to induce vascular relaxation. Nebivolol suppresses autophagy and enhances the levels of superoxide dismutase and catalase^38^. When nebivolol is administered, there was a reduction of ROS and O_2_ concentration in endothelial cells exposed to oxidative stress^39^.

Oxidative stress has been reported in reproductive toxicity^40^. All these findings together support the hypothesis that deltamethrin induced oxidative stress in the ovaries may affect ovarian dysfunction. Therefore, it is necessary to understand ovarian deltamethrin toxicity because the application of pesticides to animals and the consumption of foods containing deltamethrin may affect human health. Ovarian deltamethrin toxicity may lead to female infertility. This study suggests that nebivolol, a selective β-blocker, has positive effects on ovarian toxicity originating from the insecticide deltamethrin, thanks to its anti-oxidative aspect.

## Conclusion

It was thought that deltamethrin toxicity adversely affected follicle development by inducing degeneration and apoptotic process in preantral and antra follicle cells, and nebivolol administration might reduce inflammation and slow down the apoptotic signal in the nuclear phase and regulate reorganization.

## Data Availability

All data sets were generated or analyzed in the current study.
